# Identifying tumor antigens and immuno‐subtyping in colon adenocarcinoma to facilitate the development of mRNA vaccine

**DOI:** 10.1002/cam4.4846

**Published:** 2022-05-20

**Authors:** Huaicheng Tan, Ting Yu, Chunhua Liu, Yang Wang, Fangqi Jing, Zhenyu Ding, Jiyan Liu, Huashan Shi

**Affiliations:** ^1^ Department of Biotherapy, Cancer Center, West China Hospital, Sichuan University Chengdu China; ^2^ Department of Pathology and Laboratory of Pathology, State Key Laboratory of Biotherapy, West China Hospital, West China School of Medicine, Sichuan University Chengdu China; ^3^ State Key Laboratory of Oral Diseases, National Clinical Research Center for Oral Diseases, West China Hospital of Stomatology, Sichuan University Chengdu China; ^4^ Department of Radiotherapy Cancer Center and State Key Laboratory of Biotherapy, West China Hospital, Sichuan University Chengdu China

**Keywords:** colon adenocarcinoma, immune landscape, immune subtypes, mRNA vaccine, tumor antigens

## Abstract

The mRNA vaccine has provided a promising approach for cancer immunotherapies. However, only a few mRNA vaccines have been developed against colon adenocarcinoma (COAD). Screening potential targets for mRNA vaccines from numerous candidates is a substantial challenge. Considering the tumor heterogeneity, only a subset of patients might respond to vaccinations. This study was conducted to identify potential candidates for mRNA vaccines, and distinguish appropriate subgroups of COAD patients for vaccination. A total of five tumor antigens with prognostic values were identified, including IGF2BP3, DPCR1, HOXD10, TRIM7, and ZIC5. The COAD patients were stratified into five immune subtypes (IS1‐IS5), according to consensus clustering analysis. Higher tumor mutation burden (TMB) was observed in IS1 and IS5 subtypes. The IS1 and IS5 subtypes have shown the baseline of immune‐hot tumor microenvironment, while other subtypes displayed immune desert phenotype. Distinct expressions of immune checkpoints (ICPs)‐related genes and immunogenic cell death (ICD) modulators were observed among five immune subtypes. Finally, the immune landscape was conducted to narrow the immune components for better personalized mRNA‐based vaccination. The IFIT3, PARP9, TAP1, STAT1, and OAS2 were confirmed as hub genes, and COAD patients with higher expressions of these genes might be more appropriate for mRNA vaccination. In conclusion, the IGF2BP3, DPCR1, HOXD10, TRIM7, and ZIC5 were identified as potential candidates for developing mRNA vaccines against COAD, and patients in IS1 and IS5 subtypes might respond better to mRNA vaccination.

## Graphical abstract

Selecting appropriate neoantigens with clinically relevant and immunogenic remains key issue for developing effective mRNA vaccines. By screening the aberrantly expressed and mutational profiles of COAD samples, five tumor antigens with prognostic and immune‐infiltrating relevance were identified as promising candidates for mRNA vaccine. The COAD patients were further stratified into five immune subtypes based on immune‐related gene profiles, among which the IS1 and IS5 subtypes with higher TMB, somatic mutation rates and inflamed tumor microenvironment might benefit more from mRNA vaccination. This study not only offers a theoretical foundation of developing mRNA vaccines and screening appropriate COAD subsets for vaccination, but also provides a scientific method for identifying potential antigens of vaccines in other cancer types or even pandemics.

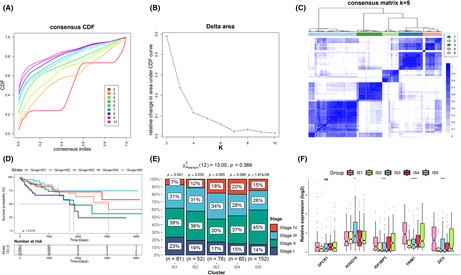

## 1 INTRODUCTION

As one of the most common causes of cancer‐related death worldwide, the colon adenocarcinoma (COAD) has become a major public health problem.^1^ The occurrence of COAD is often due to the accumulation of mutational burden, especially the mutation of tumor suppressor genes and the activation of tumor‐promoting genes.^2^ The current treatment strategies for COAD mainly include surgery, in combination of chemotherapy or radiotherapy, targeted therapy, and immunotherapy.^3^ Although the overall survival (OS) has been double prolonged for patients with non‐metastatic COAD, the survival of advanced COAD is still unsatisfactory.^3^ The systemic toxicity and adverse reactions of chemotherapy, and innate or acquired resistance of targeted therapy limit the outcomes of COAD. Patients who have accepted with surgery or chemotherapy usually would undergo recurrence or metastasis.^4^ Recently, a tumor vaccine targeting guanylyl cyclase C (GUCY2C) (NCT01972737) has shown well immunological efficacy and safety.^5^ Meanwhile, the cancer vaccines targeting carcinoembryonic antigen (CEA) (NCT01890213), melanoma associated antigen (MAGE) (NCT00020267), and RAS (NCT00019006) for COAD are ongoing. Although not all clinical results are available, these results are still encouraging enough for exploring the great potential of cancer vaccines.

The typical cancer vaccines involve in exogenously administrating tumor antigens with adjuvants, which could be delivered to antigen‐presenting cells (APCs), predominantly dendritic cells (DCs), inducing strong CD4^+^ T cells and cytotoxic T lymphocytes (CTLs) responses to eliminate tumor cells.^6^ The ideal cancer vaccines could induce tumor regression, eliminate residual tumor cells, and generate sustained anti‐tumor immunity without obvious side effects.^6^ According to antigen formats, cancer vaccines mainly include DCs vaccines, peptide vaccines, viral vectors, and genetic vaccines (DNA or RNA).^7^ The feasibility of mRNA‐based cancer vaccines could be traced back to 1990 and rapidly develop into an attractive form of cancer vaccines.^8^ mRNA vaccines encode tumor‐associated antigens (TAAs), like overexpressed antigens or tumor‐specific antigens (TSAs) such as mutated neoantigens, which further induce delivery of antigen and co‐stimulation mediated by innate immune independent of priorly identifying human leukocyte antigen (HLA) haplotype or predicting epitope.^9^ mRNA vaccines have shown various advantageous features over peptides, tumor cells, and DNA‐based vaccines. Firstly, the mRNA is biodegradable, and its half‐time could be adjusted by modifying the RNA sequence or using suitable delivery systems. The proteins or peptides encoded by mRNA application are rapid and short‐lived with a duration of several days, which makes the mRNA vaccines controllable and safe.^10^ In addition, the DNA sequences might integrate into the genome of tumor cells and result in potential insertional mutagenesis, while mRNA has no risk of gene integration or gene deletion.^11^ Unlike the time and resource consuming characteristics of DC vaccines, the development of mRNA vaccines is faster and more flexible.^12^ The versatile mRNA platform could adapt to various targets, which further allows the rapid development of vaccines, especially for newly emerging pathogens. For instance, the BNT162b2, a type of COVID‐19 mRNA vaccine, has completed the phase II/III clinical trials and been approved for usage in several countries.^13^ The lessons learned from the development of COVID‐19 vaccine have acted as leverage to accelerate the progress of mRNA‐based cancer immunotherapy. Therefore, the mRNA vaccines have provided a promising future for anti‐cancer approaches. Several mRNA vaccines, with or without adjuvants in monotherapy or combination therapy forms, have successfully stimulated the immune response in breast cancer (MUC1 mRNA), prostate cancer (CV9103), melanoma (FixVac or BNT111), and non‐small cell lung cancer (CV9201).^14^, ^15^, ^16^

Only a few mRNA vaccines have been developed against COAD so far, while the efficacy is still beyond satisfied.^17^, ^18^ Featured with high tumor‐specificity and abundance in various tumor types, the mutated neoantigens have rapidly become promising vaccine targets.^19^ The neoantigen‐specific T cells are more likely to stimulate high‐affinity T‐cell responses without causing central tolerance, whereas the TAAs might face with breaking tolerance. However, the COAD still lacks appropriate targets for mRNA vaccines, due to the screening and identification of potential neoantigens for mRNA vaccines from numerous candidates is a substantial challenge. Furthermore, considering the tumor heterogeneity and diversity of tumor immune microenvironment, only a subset of COAD patients might respond to mRNA vaccines.^20^ Accordingly, this study was performed to investigate the potential candidates for mRNA vaccines against COAD, and then screen appropriate subgroups for vaccination through mapping the immune landscape of COAD patients. A total of five candidate genes for mRNA vaccine were identified from the pool of mutated and overexpressed genes of COAD, which were significantly correlated with worse survival and increasingly intratumoral infiltration of APCs and T cells. The COAD patients were separated into five immune subtypes, which displayed diverse molecular, cellular, and clinical traits. Furthermore, the immune gene co‐expression modules and hub genes were identified through analyzing related gene signatures in individual patients. In general, this study not only provided scientific and reliable methods for identifying targets in developing mRNA‐based cancer vaccines, but also demonstrated the immune landscape of COAD and further screened the appropriate subgroups for vaccination. The workflow of this study is illustrated in Figure S1.

## 2 MATERIALS AND METHODS

### 2.1 Data extraction and data processing

The RNA‐Seq data and corresponding clinical information of 422 COAD patients were extracted from The Cancer Genome Atlas (TCGA, https://www.cancer.gov/tcga) by using the GDC Application Programming Interface (API). First, the tumor samples lacking relevant clinical infromation were excluded. The genes with 0 Fragments Per Kilobase of transcript per Million mapped reads (FPKM) in over 50% of the samples were also excluded. Then, the gene expression data of FPKM‐normalized were converted to Transcripts Per Million reads (TPM) normalized for subsequent analysis. Finally, a total of 2212 immune‐related genes were extracted, including antigen processing and presentation‐related, T‐cell receptor (TCR) signaling pathway‐related, cytokines‐related, cytokine receptors‐related and other immune‐related genes. Moreover, the datasets of GSE14333 and GSE17536 with 403 COAD patients based on the platform GPL570 were downloaded. (https://www.ncbi.nlm.nih.gov/geo/query/acc.cgi). The “limma” and “sva” R packages were applied to normalize the raw data of the gene expression and batch effect correction about the expression data in GSE14333 and GSE17536 datasets, respectively. Owing to the lack of mutation data in GSE14333 and GSE17536 datasets, these two datasets were partly used as validation cohorts.

### 2.2 Mutational pattern

The mutational signatures of cancer identified by Alexandrov et al. were obtained from the http://cancer.sanger.ac.uk/cosmic/signatures.^21^ The MuTect2‐identified mutation data containing 399 COAD specimens from TCGA was downloaded using the TCGAbiolinks package. To predict suitable clusters, the consensus‐based Non‐negative matrix factorization (NMF) was used to stratify the mutational information based on the mutational spectra of 96‐trinucleotide though R package “NMF.”^22^ Three predominant signatures of mutagenesis were identified according to optimum K value. The R package “MutationalPatterns” was used for displaying the mutational landscape of all COAD samples.

### 2.3 Validation of mutational signature

The weight of each mutational signature in an individual sample was computed by using R package “deconstructSigs”, with the signature contribution cutoff of 6%.^23^ According to the weight of each mutational signature, the consensus NMF clustering was performed with optimum K value to recognize the subgroup of mutational signatures. The optimum K value was determined where the highest Cophenetic coefficient was exhibited.

### 2.4 Identification of potential tumor antigens

By using the R package “survminer”, the optimal cut‐off for mutational signature score was determined based on the relationships between OS of patients and current gene expression level in individual data set. According to the threshold, the patients were divided into the low and high‐score groups in order to evaluate the prognostic value of corresponding mutational signatures. Next, the R package “limma” was used to identify the differentially expressed genes (DEGs) between the low‐score and high‐score groups. The DEGs with a |log2 fold change (FC)| > 1 and false discovery rate (FDR) < 0.05 were screened for subsequent analysis. The Kaplan–Meier survival curve was drawn to evaluate the associations between the DEGs expression levels and OS based on the optimal cut‐off. The DEGs associated with unfavorable prognosis were further selected as candidate antigens. The mutational frequencies of candidate antigens in COAD samples from TCGA cohort were analyzed by using the “maftools” R package.^24^ The genes with mutational frequencies over 1% were identified as potential antigens.

### 2.5 Evaluating the immune cell infiltration through TIMER and antigens quality scoring

Tumor Immune Estimation Resource (TIMER) web (https://cistrome.shinyapps.io/timer/) provides a comprehensive resource for investigating the correlations between tumor‐infiltrating immune cells and multiple factors such as gene expression levels.^25^ Therefore, the associations between the expression levels of identified potential antigens and infiltration levels of immune cells were calculated and visualized by using the gene module of TIMER. Besides, the purity adjustment was selected in gene module, which applied the partial Spearman's correlation to implement the association analysis of gene expressions and immune infiltration. Furthermore, the potential of these candidate antigens for peptide vaccines was also evaluated by using the Immune Epitope Database (IEDB).^26^ Based on the similarity of the imported amino acid sequences of corresponding antigens to the human infectious disease‐derived peptide sequences with positive immune assays from IEDB, the probability that the antigen might be recognized by the T cell receptors (TCRs) were evaluated. Consistent with previously used filtering criterion, the peptides from candidate antigens with predicted major histocompatibility complex (MHC) binding affinity below 500 nM were retained as neoantigens.^27^

### 2.6 Identification of the immune subtypes and corresponding prognostic evaluation

According to the expression profiles, a total of 2212 immune‐related genes were clustered, followed by constructing a consensus matrix to demonstrate relevant immune subtypes and gene modules. Besides, the partition around medoids (PAM) algorithm was adapted with “Pearson correlation” based distance metric. The bootstraps with 1000 iterations were implemented, with each iteration involving 90% of the samples. The optimal clustering pattern was identified based on the cumulative distribution function (CDF) curves of the consensus score and heatmap of consensus matrix. Furthermore, the Kaplan–Meier method was used to draw survival curves, and the prognostic significance of the immune subtypes was assessed through log‐rank test. In addition, referring to previously reported signature gene sets, the immune contexture feature for each sample in immune subtypes was also calculated by using the single‐sample Gene Set Enrichment Analysis (ssGSEA).^28^

### 2.7 Analysis of immune‐related molecular and cellular characteristics

To investigate the pathways correlated with the immune‐related molecular and cellular characteristics, the R package “clusterProfiler” was employed to analyze the enrichment analysis of the Gene Ontology biological process (BP). Next, the correlations between the various immune subtypes of COAD and 56 previously established immune‐related molecular characteristics were evaluated.^28^

### 2.8 Construction of immune landscape

To better visualize the distribution of individual patients, the graph learning‐based dimensionality reduction analysis was applied to analyze the immune‐related gene expression profiles.^29^ The reduced dimension function of the “Monocle” package with a Gaussian distribution was employed during this process. With setting the maximum number of components at two, the dimension reduction was conducted by using the discriminative dimensionality reduction with trees (DDRTree). Subsequently, the function plot cell trajectory (package “Monocle”) was performed for the visualization of immune landscape, which coded with a color corresponding to the previously defined immune subtype.

### 2.9 Statistical analysis

All statistical analyses in this study were conducted by using the R software (version 4.0.4). The used R packages were described in the corresponding steps. For comparison among multiple groups, the Kruskal–Wallis test was performed, and the Wilcox test was applied for comparison between two groups. The correlations between categorical variables were analyzed by using the chi‐square test. The statistical significance was considered with a p value less than 0.05.

## 3 RESULTS

### 3.1 Identification of the mutational signature profiles in COAD

**FIGURE 1:**
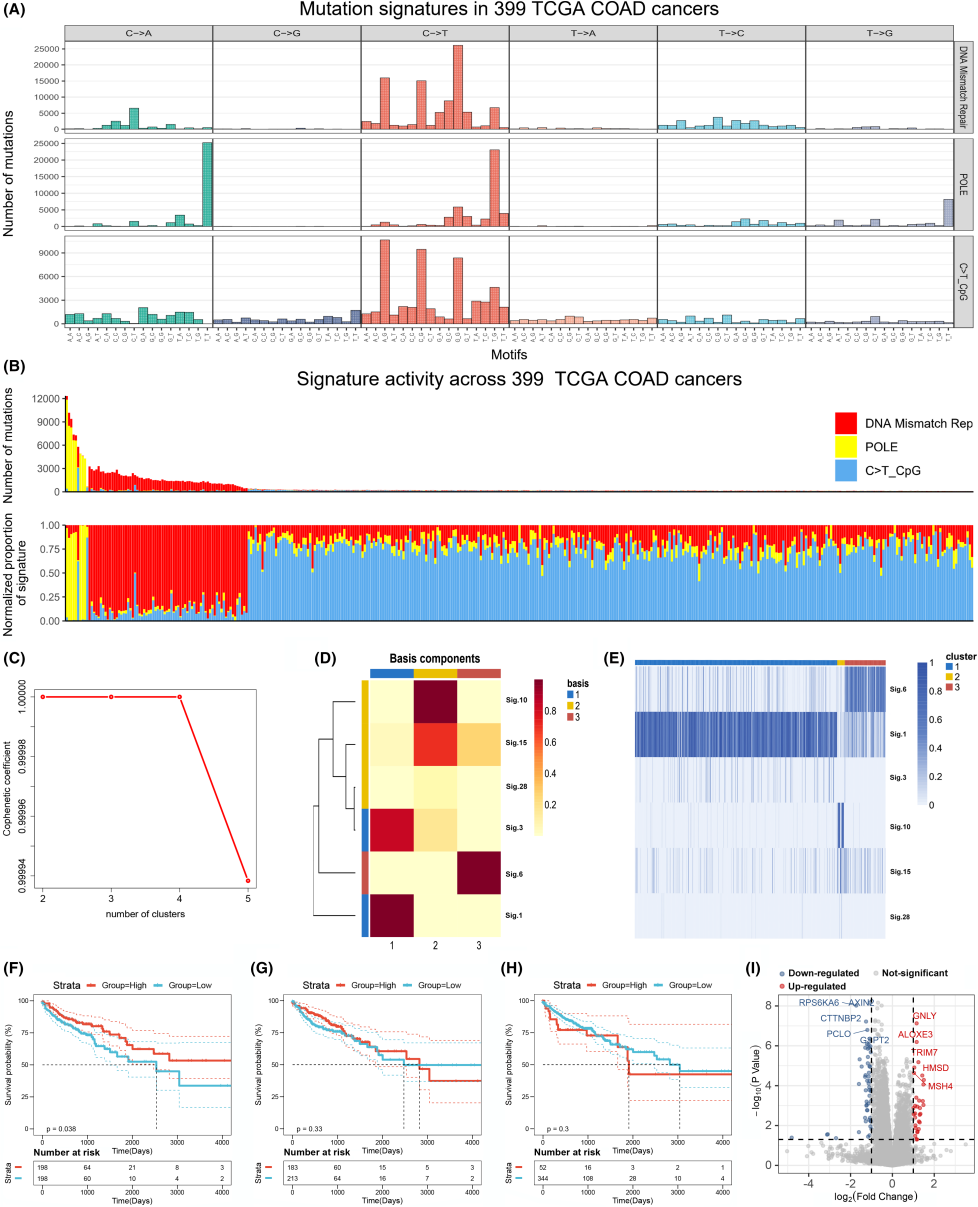
Mutation signatures and molecular classification of COAD samples in TCGA cohort. (A) The Bayesian NMF was applied to analyze the mutational signatures through clustering mutational information in COAD, of which three mutational signatures including DNA mismatch repair, POLE, and C > T mutations at CpG islands (C > T CpG) were identified. (B) The number of mutations about three mutational signatures and relevant proportions of signatures were analyzed in individual COAD samples from TCGA cohort. (C) Identifying the number of clusters by cophenetic coefficient. (D) The basic components of NMF with *k* = 3. (E) The clustering analysis revealed three cluster groups based on distinct mutational signatures. The Kaplan–Meier curves revelated the prognostic values of the mutational signatures attributed to DNA mismatch repair (F), POLE (G), and C > T CpG (H) in COAD patients from TCGA cohort. (I) The volcano plot illustrated the top 10 DEGs in COAD patients with high or low scores of mutational signatures attributed to DNA mismatch repair.

As novel proteins, the neoantigens are most commonly the consequences of somatic mutations.^30^ The somatic mutations are the results of multiple mutational processes, which generate distinct combinations of mutation types known as mutational signatures.^21^ To screen potential antigens of COAD, the Bayesian NMF analysis was applied to analyze the mutational signatures through stratifying 96 tri‐nucleotide contexts in 399 TCGA COAD samples (Figure 1A and Figure S2). Three predominant signatures of mutagenesis were identified in COAD, including the DNA mismatch repair, POLE (DNA polymerase epsilon catalytic subunit), and C > T mutations at CpG islands (C > T CpG). Besides, the number of mutations about the three mutational signatures is diverse among individual COAD samples (Figure 1B). To better characterize COAD, the molecular classification based on mutational signatures was performed. According to the Cophenetic coefficient defined in Brunet et al, the number of clusters was set at 3 (Figure 1C).^31^ The basic components of NMF with *k* = 3 was shown in Figure 1D. Three distinct clusters could be observed in consensus matrix based on the relative distribution of three mainly mutational signatures of COAD (Figure 1E). For exploring the prognostic value of these mutational signatures in COAD, we further divided the cohort samples into high score and low score groups according to the individually optimum cutoff values. Patients with higher score in mutational signature of DNA mismatch repair (signature 1) were significantly related to better survival, while no significant differences were found between the high and low score groups from the mutational signatures of the POLE (signature 6) and C > T CpG (signature 10) (Figure 1F–H). Therefore, the DEGs between patients with high and low scores of DNA mismatch repair were further illustrated, of which the top 10 regulated‐genes were displayed in the volcano plot (Figure 1I). Taken together, a total of 64 DEGs in signature of DNA mismatch repair in COAD samples were identified for subsequently screening potential antigens.

### 3.2 Identifying tumor antigens relevant to prognosis and anti‐tumor immunity in COAD

**FIGURE 2:**
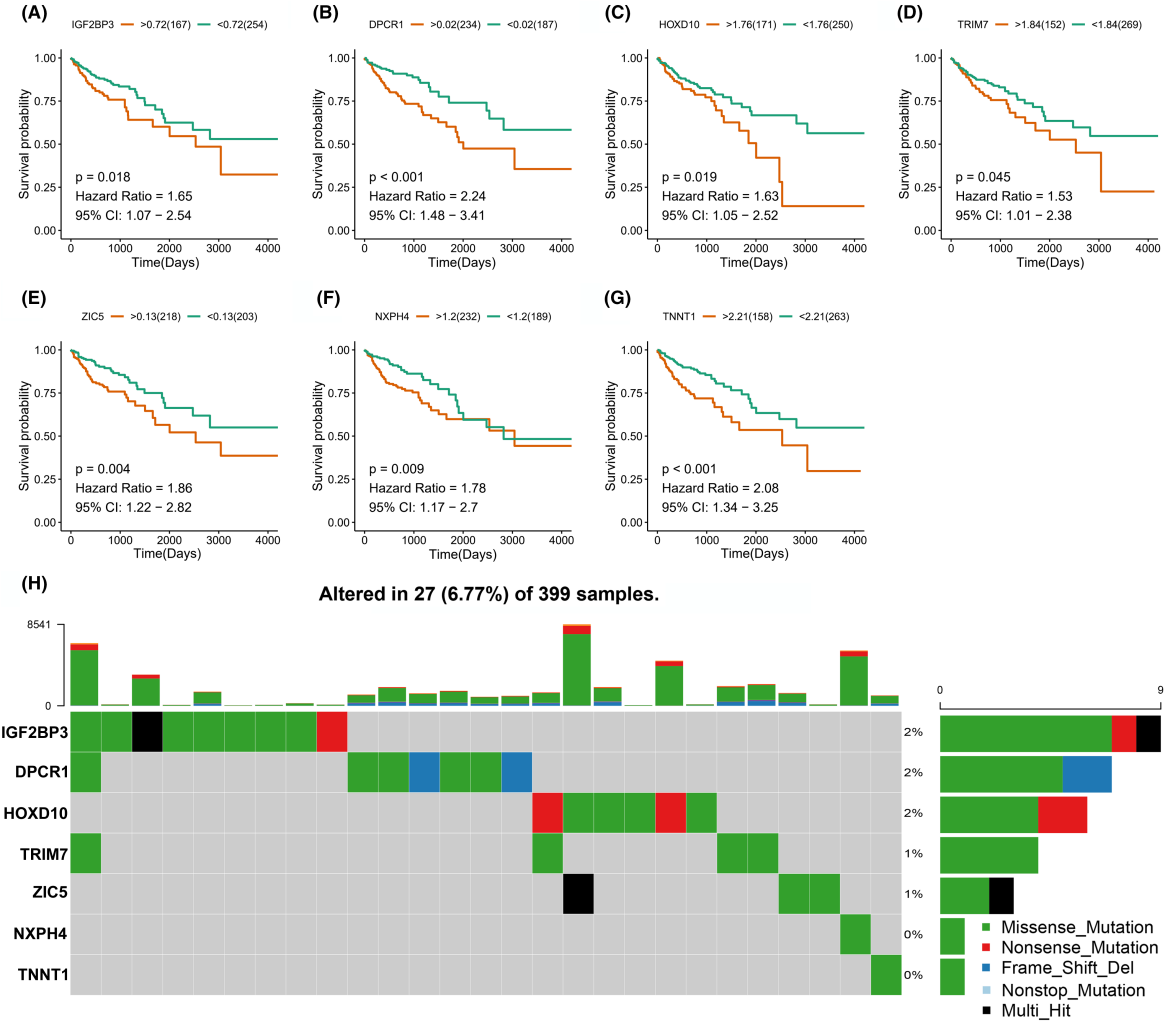
Prognostic relevance and mutation status of the potential tumor antigens. The higher expression levels of candidate genes for mRNA vaccine, including IGF2BP3 (A), DPCR1 (B), HOXD10 (C), TRIM7 (D), ZIC5 (E), NXPH4 (F), and TNNT1 (G) were associated with worse OS in COAD patients, as calculated by the Kaplan–Meier analysis. (H) The oncoplot showed the mutations of the 7 prognostically relevant genes in COAD samples.

Although not all mutations could form neoantigens, more tumor antigens will be generated when a tumor has more mutations.^32^ Accordingly, higher tumor mutation burden (TMB) has been gradually regarded as a potential biomarker for recognizing tumor antigens.^33^ The prognostically relevant tumor antigens were further screened from the pool of DEGs. As shown in Figure 2A–G, a total of 7 genes were significantly associated with the prognosis of COAD patients. The patients with higher expression levels of insulin like growth factor 2 mRNA binding protein 3 (IGF2BP3), Diffuse panbronchiolitis critical region 1 (DPCR1), homeobox D10 (HOXD10), Tripartite Motif Containing 7 (TRIM7), Zic Family Member 5 (ZIC5), neurexophilin 4 (NXPH4), and troponin T1 (TNNT1) were significantly correlated with shorter OS in comparison with those in low‐expression groups. Subsequently, the mutations of these 7 genes in COAD patients were analyzed by using the MuTect2‐processed mutation dataset in TCGA, which is shown in the oncoplot (Figure 2H). The genes with mutational frequency higher than 1%, including IGF2BP3, DPCR1, HOXD10, TRIM7, and ZIC5 were further selected as the potential candidates. As validated in the datasets of GSE14333 and GSE17536, the COAD patients with higher expression levels of these five genes were also significantly with worse survival (Figure S3). As the central factors in developing the anti‐tumor vaccine, the tumor antigens could be processed by APCs for further priming of T cells and activating anti‐tumor immunity.^34^ Analysis of tumor immune infiltration showed the elevated expression levels of IGF2BP3, DPCR1, HOXD10, TRIM7, and ZIC5 tended to be correlated with the increased tumor infiltrating B cells, CD8^+^ T cells, CD4^+^ T cells, and APCs including macrophages and DCs in COAD patients (Figure S4). The therapeutic potential of mRNA vaccines are depended on triggering of immunity by their encoded proteins or peptides.^35^ Therefore, the MHC binding probability of the peptides from IGF2BP3, DPCR1, HOXD10, TRIM7, and ZIC5 were predicted by using the IEDB. As shown in Table S1, these five candidate antigens all demonstrated high MHC binding affinity, which further supported their therapeutic potential. Taken together, these results suggested the prognosis‐related tumor antigens including IGF2BP3, DPCR1, HOXD10, TRIM7, and ZIC5 were promising targets of being developed as mRNA vaccines against COAD.

### 3.3 Identifying immune subtypes of COAD

**FIGURE 3:**
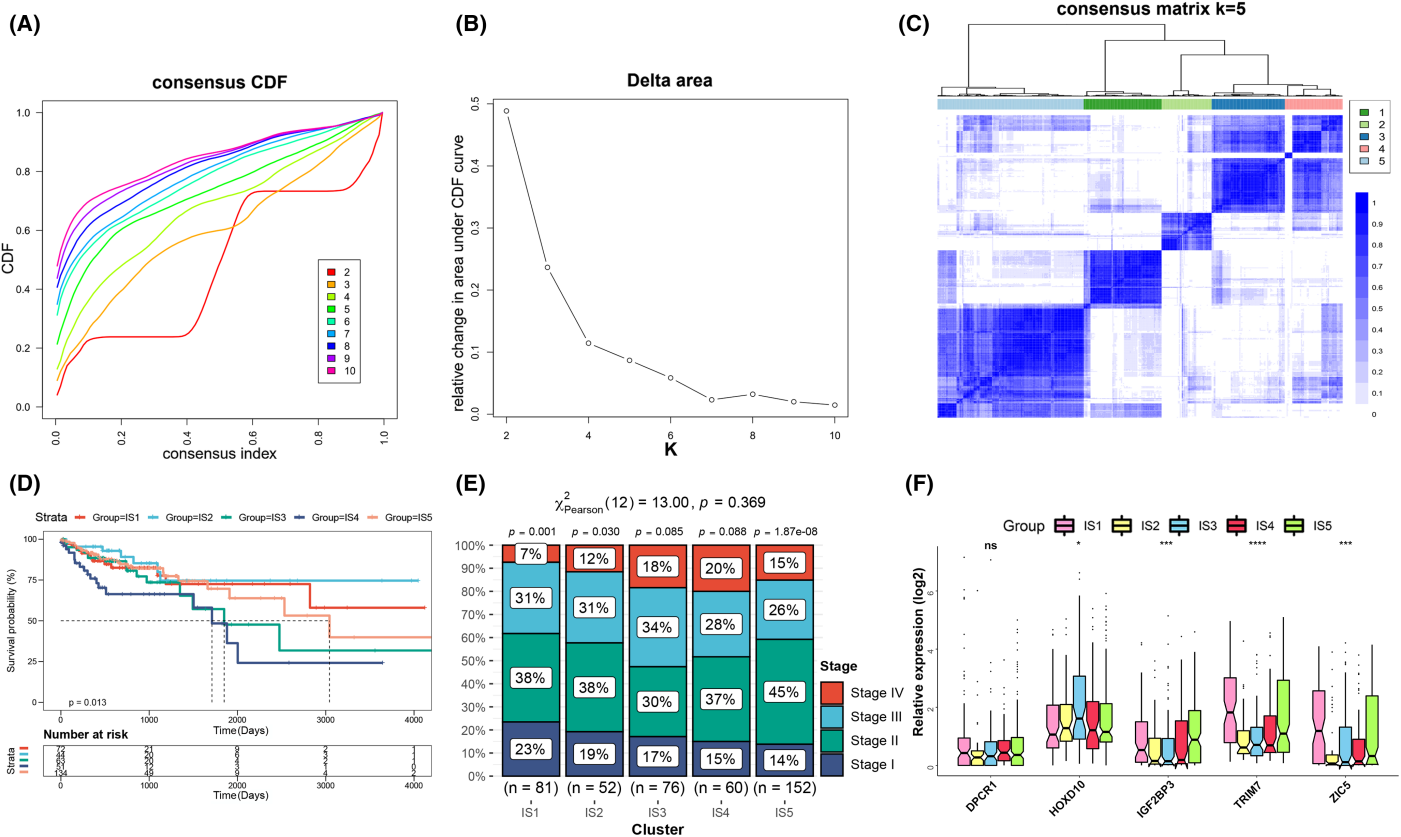
Immunosubtyping of COAD. The CDF curves (A) and delta area of immune‐related genes (B) were analyzed in TCGA cohort. (C) Clustering heat map of COAD based on immune‐related gene expression profile in TCGA cohort. (D) OS analysis of COAD patients with different immune subtypes in TCAG cohort. (E) Staging distribution in COAD patients with IS1‐IS5 in TCGA cohort. (F) The expression levels of the five candidates in the distinct immune subtypes

To better assess the immune status in tumors and screen appropriate patients for vaccination, we next constructed the consensus clustering based on the immune‐related gene expression profile in 364 COAD samples form TCGA cohort. The *k* = 5 where the immune‐related genes tended to be stably clustered was determined, according to the CDF and function delta area (Figure 3A,B). A total of 5 immune subtypes for COAD patients were identified and assigned as IS1‐IS5 (Figure 3C). As shown in Figure 3D, the IS3 and IS4 were relevant to worse survival, while patients in IS1, IS2, and IS5 appeared to have better prognosis. Furthermore, the staging distributions of COAD patients in different immune subtypes were also exhibited in Figure 3E. In accordance with the survival, the IS3 and IS4 subtypes showed higher proportions of Stages III and IV. Besides, the expression levels of the five candidates including IGF2BP3, DPCR1, HOXD10, TRIM7, and ZIC5 were investigated in the distinct immune subtypes, among which the IS1 and IS5 subtypes have shown relatively higher expression levels of these candidates than other subtypes (Figure 3F). Moreover, five immune subtypes for COAD patients from datasets of GSE14333 and GSE17536 were also identified through consensus clustering based on immune‐related gene expression profile. The patients in the IS4 subtype have shown obviously worst survival (Figure S5). Taken together, these results indicated the COAD patients could be potentially identified as five immune subtypes with prognostic relevance according to the clustering of immune‐related gene expression profile.

### 3.4 Assessment of the mutational status in COAD immune subtypes

**FIGURE 4:**
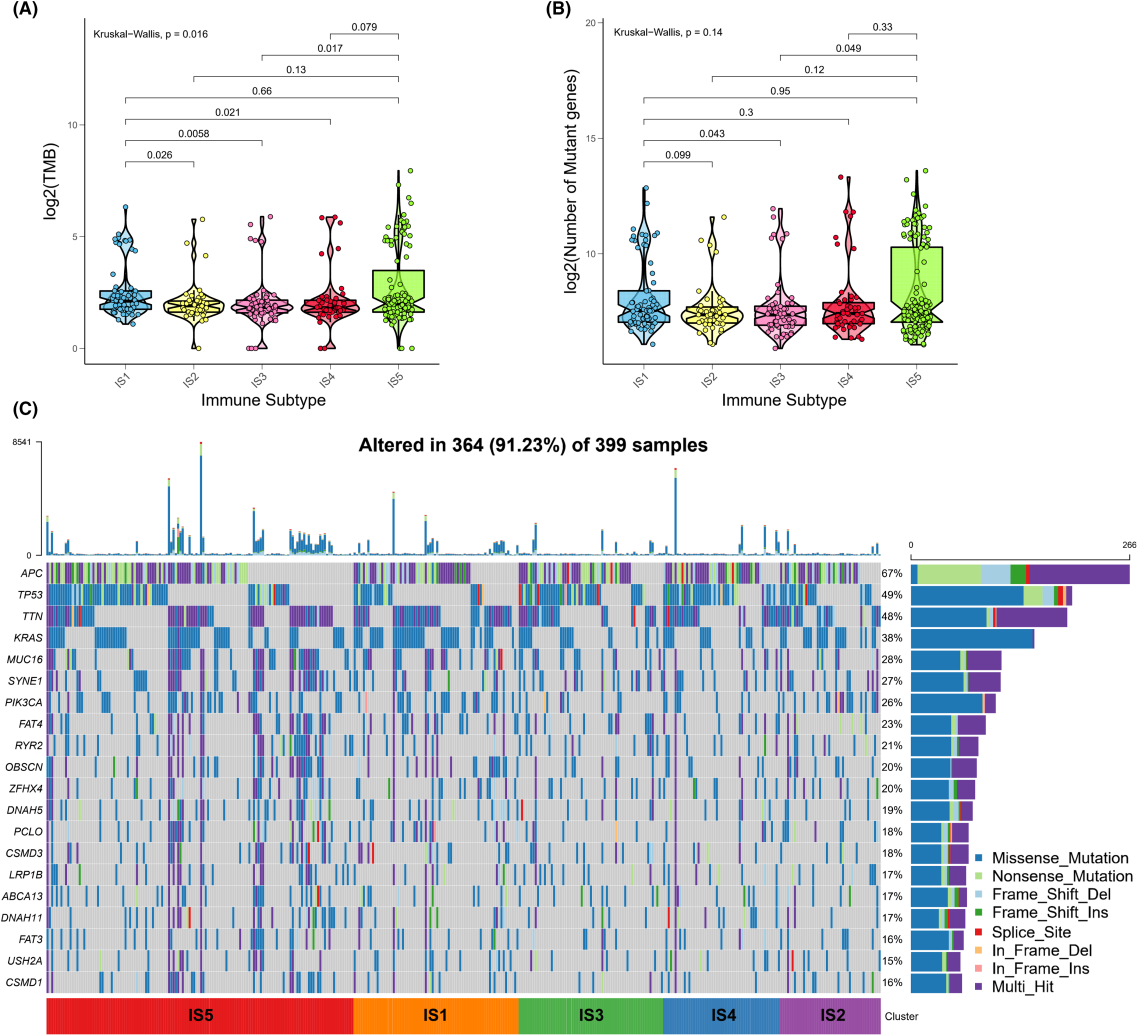
Comparisons of the TMB and mutation in COAD immune subtypes. The TMB (A) and the number of mutated genes (B) were assessed in five immune subtypes of COAD. (C) The landscape of 20 genes with the most frequently genomic alteration in COAD immune subtypes.

More than being used as biomarker for identifying neoantigen, the higher TMB in tumors is also closely associated with better immunotherapeutic responses.^36^ Hence, the TMB and number of mutated genes in distinct immune subtypes were calculated based on the MuTect2‐processed mutation dataset in TCGA. Significantly higher TMB was observed in IS1 and IS5 subtypes in comparison with that in IS2, IS3 and IS4 subtypes (Figure 4A). Besides, the IS1 and IS5 subtypes also showed relatively higher numbers of mutated genes (Figure 4B). Moreover, the landscape of 20 genes with most frequently genomic alteration was also assessed in five immune subtypes, which displayed obviously genomic alteration in IS1 and IS5 subtypes (Figure 4C). Collectively, the various immune subtypes of COAD exhibit diversely mutational status, in which patients of IS1 and IS5 subtypes are more like to respond to immunotherapy. Limited by lacking of mutation data in other datasets of COAD, the mutational status of COAD immune subtypes was only analyzed in the TCGA cohort.

### 3.5 Expression status of immune modulators in CDAD immune subtypes

Based on the functional pattern of mRNA vaccine, the efficacy of mRNA vaccine greatly depends on the host immunity status. Acting as the gatekeeper of anti‐tumor immunity, the immune checkpoints (ICPs) negatively regulates the efficacy of immunotherapy.^37^ In relation to cancer immunotherapy, the immunogenic cell death (ICD) could increase the adjuvanticity and antigenicity from apoptotic cancer cells, and subsequently improve the clinically desirous anti‐tumor immune responses.^38^ Given the importance of ICPs and ICD in potential influence on the efficiency of mRNA vaccine, we further investigate the expression levels of ICPs and ICD modulators in COAD immune subtypes. Among 45 ICPs‐related genes, 31 (68.89%) genes were differentially expressed (Figure S6A). A total of 12 (26.67%) ICPs‐related genes including CD244, CD27, CD274, CD40LG, CD70, CTLA4, ICOS, TIGIT, TMIGD2, TNFRSF9, TNFSF14, and VTCN1 were obviously down‐regulated in IS1 subtype in comparison with other subtypes. Besides, the lowest expression levels of ADORA2A, BTNL2, CD160, CD200, CD28, ICOSLG, KIR3DL1, NRP1, TNFRSF4, and TNFRSF8 were found in IS5 subtypes. However, the IS2 subtypes showed the obviously highest expressions of most ICPs‐related genes (60%). Furthermore, 25 ICD‐related genes were analyzed in TCGA cohort, of which 17 (68.00%) genes were differentially expressed in various immune subtypes (Figure S6B). Remarkably, the classical hallmarks of ICD like EIF2AK1, EIF2AK2, EIF2AK4, HMGB1, IFNW1, LRP1, and MET were overexpressed in IS1 subtype compared to other immune subtypes, and the expression levels of EIF2A and IFNAR1 tended to be up‐regulated in IS5 subtype. Moreover, the expression levels of ICPs and ICD modulators were also evaluated in the immune subtypes from datasets of GSE14333 and GSE17536 (Figure S7). Taken together, the various immune subtypes showed distinct expression status of ICPs and ICD modulators, which could act as biomarkers for evaluating the potential immune responses for mRNA vaccine.

### 3.6 Tumor immunological analyses in CDAD immune subtypes

**FIGURE 5:**
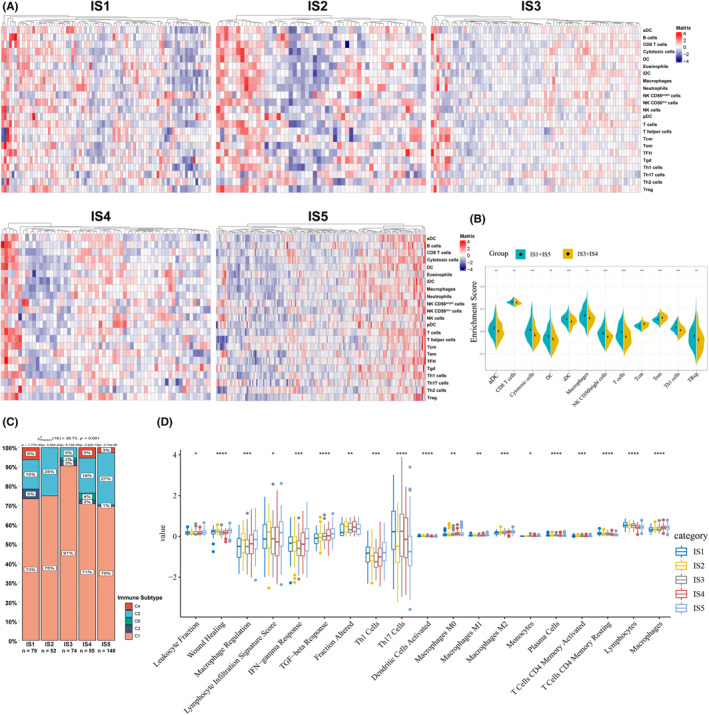
Tumor immunological analyses in COAD immune subtypes. (A) Heatmaps of the immune cell signatures in COAD immune subtypes. (B) Violin plot showed the differential enrichment scores of the immune cell signatures in various subtypes. (C) Distributions of the 6 pan‐cancer immune subtypes. (D) Immune‐related molecular features in COAD immune subtypes. Abbreviations: aDC, activated DC; iDC, immature DC; pDC, plasmacytoid DC; Tcm, central memory T cells; Tem, effector memory T cells; TFH, T follicular helper cells; Tgd, gamma delta T cells. (*P < 0.05, **P < 0.01, ***P < 0.001 and ****P < 0.0001).

The pre‐existing infiltration of immune cells in tumors is closely relevant to the treatment response and survival across different cancer types.^39^ Therefore, we next identified the immune contexture features in various COAD immune subtypes in TCGA cohort by using ssGSEA according to previously reported signature gene sets.^28^ The IS1 and IS5 subtypes demonstrated similar scores of the immune cell components, whereas the distribution in IS3 subtypes was resembled to that in IS4 subtype (Figure 5A). We further analyzed enrichment scores in these subtypes, which exhibited obviously differential enrichment scores (Figure 5B). The IS1 and IS5 subtypes showed significantly higher scores in DCs, macrophages, CD8^+^ T cells, cytotoxic cells, CD56^bright^ natural killer (NK) cells, and Th1 cells, and regulatory T cells (Tregs) when compared with those in IS3 and IS4 subtypes. Consequently, the IS1 and IS5 subtypes were characterized by higher immune infiltration phenotypes, whereas IS3 and IS4 were manifested with relatively lower immune infiltration.

Furthermore, six stable immune categories (C1‐C6) with distinct distribution of immune signatures have been identified based on analyzing more than 10,000 tumor samples involving 33 cancer types.^28^ These immune categories were closely related to the prognosis, genetic, and immune modulatory alterations, which could improve the understanding of tumor immune microenvironment and prediction of the response to immunotherapy. Therefore, we further analyzed the distributions of these six immune categories in COAD immune subtypes, in which the COAD mainly included the C1, C2, C3, C4, and C6 subtypes.^28^ Similar distributions were observed in IS1 and IS5 subtypes, which were mainly occupied by C1, C2, C3, and C4. The IS2 subtype was occupied by C1 and C2, and the IS3 subtype was mainly occupied by C1 (Figure 5C). The C6 that is relevant to worse prognosis was only distributed in the IS3 and IS4 subtypes. These results were coincident with the relatively worse survival observed in IS3 and IS4 subtypes. Accordingly, these findings further supported the reliability of our immune typing manner.

Next, the correlations of the various COAD subtypes and 56 previously established immune‐related molecular signatures were explored, and 19 significant signatures were recognized. The IS1 was featured with relatively high scores in Th1 cells, DCs activated, CD4^+^ T cells memory resting, and lymphocytes, with lowest score in TGF‐β response. The IS5 was characterized by highest scores in leukocyte fraction, macrophage regulation, INF‐γ response, M1 macrophage, and CD4^+^ T cells memory activated compared with other subtypes, with lowest scores in Th17 cells (Figure 5D). In contrast, the IS3 and IS4 subtypes displayed relatively lower scores in leukocyte fraction, lymphocyte infiltration signature score, IFN‐γ response, and Th1 cells. The higher immunological score or more tumor‐infiltrating immune cells before vaccination has been reported to be positively associated with response to cancer vaccines.^40^, ^41^ Therefore, the various immune subtypes of COAD displayed distinctly immunological status, in which patients of IS1 and IS5 subtypes with higher immunological scores were potential subgroups for mRNA vaccine.

### 3.7 Immune landscape of COAD

**FIGURE 6:**
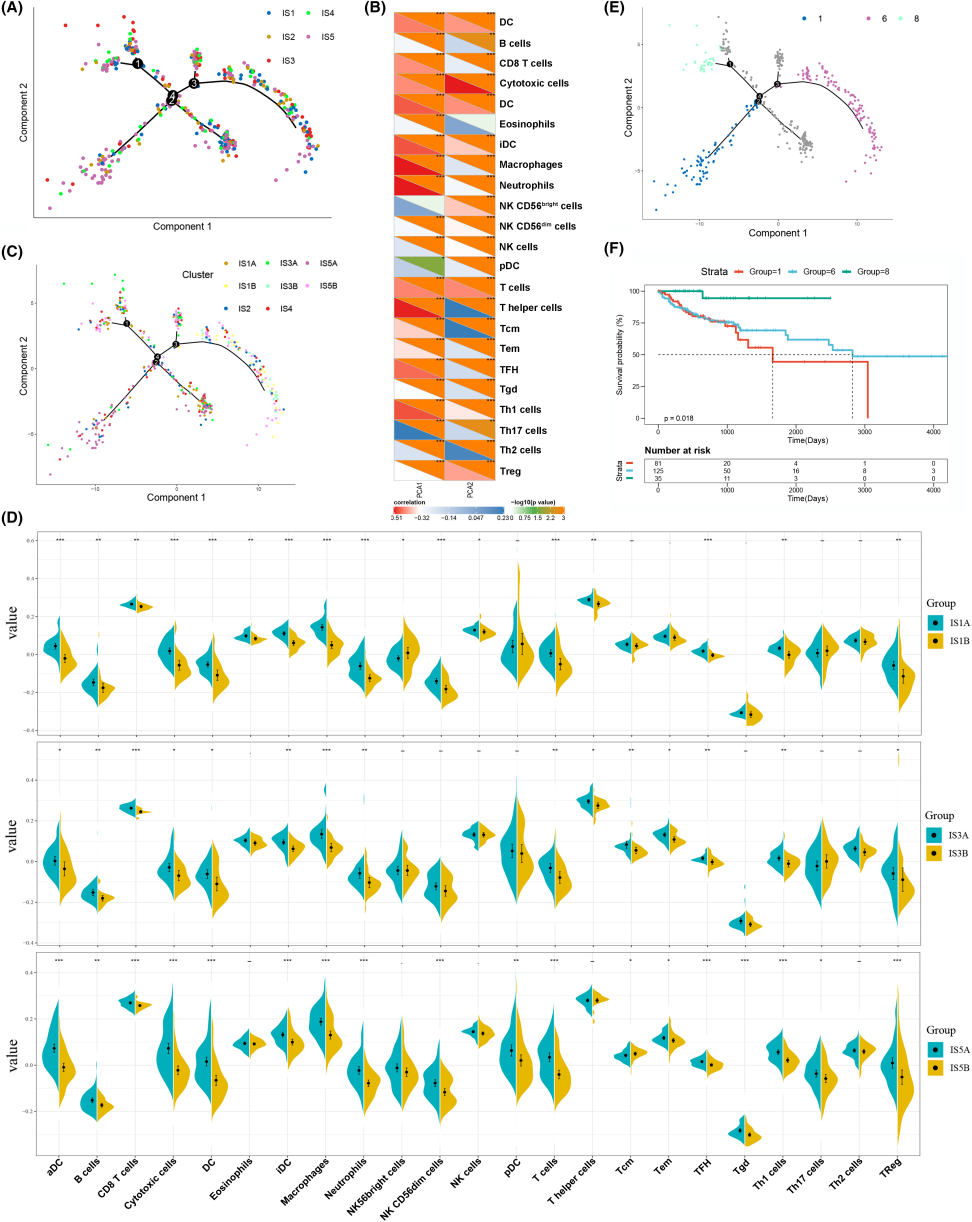
The immune landscape of COAD showed the intracluster heterogeneity. (A) The immune landscape of COAD based on dimensionality reduction analysis. Each point indicated a patient, which was coded with color corresponding to the previously defined immune subtype. (B) Heat map revealed the correlations between the principal component analysis 1/2 (PCA1/PCA2) and various immune signatures. (C) According to the locations in the immune landscape, the IS1, IS3, and IS5 could be further divided into different subsets. (D) Violin plot displayed the differential enrichment scores of the immune cell signatures in the above subsets. (E) The patients from three extreme locations as separated by the immune landscapes were related to significantly distinct outcomes (F). (*P < 0.05, **P < 0.01 and ***P < 0.001).

For better visualizing and revealing the distribution of individual patients, the immune‐related gene expression profiles were analyzed by the graph learning‐based dimensionality reduction technique. As shown in Figure 6A, the individual patients were allocated into the tree structure, and the immune landscape of COAD was constructed. The horizontal axis represented the first principal component (PCA1), while the second principal component (PCA2) was represented by the vertical axis. The PCA1 was negatively associated with most immune cells, including activated DCs, CD8^+^ T cells, cytotoxic cells, macrophages, T helper cells, Th1 cells, whereas the PCA2 was negatively associated with multiple immune cells, including CD8^+^ T cells, cytotoxic cells, DCs, macrophages, CD56^bright^ NK cells, T cells, effector memory cells (Tem), and Th1 cells (Figure 6B). Based on the immune landscape of COAD, intracluster heterogeneity was observed. The IS1, IS3, and IS5 subtypes appeared to be respectively divided into two subsets with diverse locations (Figure 6C). The subsets in IS1 and IS5 displayed obviously different enrichment scores of immune cells. For instance, the IS1A displayed significantly higher enrichment scores of DCs, macrophages, CD8^+^ T cells, cytotoxic cells, NK cells, and Th1 cells (Figure 6D). Similar higher enrichment scores of immune cells were also observed in IS5A subset in comparison with the corresponding subset (Figure 6D). Therefore, the IS1A and IS5A subsets were much more suitable for the mRNA vaccination. Moreover, the patients of three extreme locations could be identified in the immune landscapes, which were associated with distinct outcomes (Figure 6E,F). Collectively, the immune landscape analysis could further complement the previously identified immune subtypes, which is beneficial for predicting prognosis and screening suitable subsets for mRNA vaccination.

### 3.8 Enrichment analysis of immune gene co‐expression modules and identification of hub genes

**FIGURE 7:**
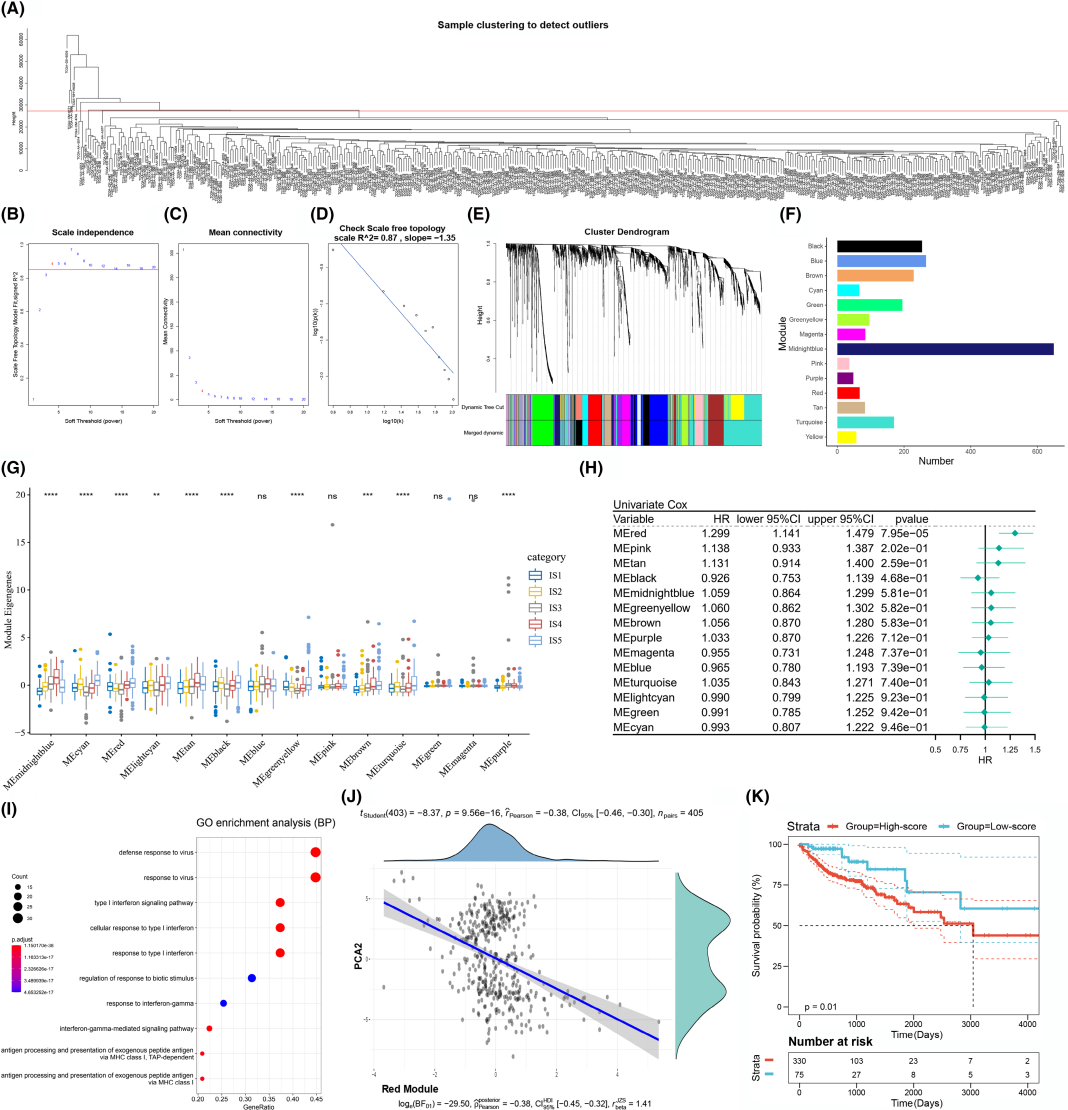
The immune gene co‐expression modules and identifying the immune hub genes of COAD. (A) Sample clustering to detect outliers. Identifying the soft‐thresholding power by analyzing the scale‐free index (B), mean connectivity (C) and checking scale‐free topology (D). (E) Cluster dendrogram of the differentially expressed genes. (F) Bar plot displayed the co‐expressed gene numbers in each module. (G) Expression levels of the various gene modules in COAD immune subtypes. (H) Forest map showed the survival analysis of 14 modules in COAD. (I) Dot plot showed the GO enrichment analysis of BP in red module. (J) Association between the feature vector of red module and PCA2. (K) Survival analysis of the red module with high or low score.

Considering the significance of immune‐related genes in influencing the efficacy of mRNA vaccine, we further constructed the immune gene co‐expression modules of COAD through WGCNA package of R software (Figure 7A). According to the analyses of the scale‐free fit index, mean connectivity, and checking scale free topology, the soft‐thresholding power was set at five so that the network could fit the scale‐free better (Figure 7B–D). The representation matrix was then converted to the adjacency matrix, following to a topological matrix. Depending on the hybrid dynamic shear tree standard, the average‐linkage hierarchy clustering method was performed for clustering genes and the minimum number of genes for each network was chosen as 30. We next calculated the eigengenes for each module, of which the closer modules were combined into a new module through setting the height as 0.25, and the deep split as four (Figure 7E). Consequently, 14 co‐expression modules were clustered and shown in various colors, while the genes that could not cluster with others were placed in gray module (Figure 7F). As shown in Figure 7G, the 10 modules displayed significantly different distributions in the five immune subtypes. The IS5 subtype showed relatively highest eigengenes in cyan, red, lightcyan, black, greenyellow, brown, and turquoise modules. Furthermore, the prognostic correlations of the various modules were shown in the forest map, in which the red module was significantly related to the prognosis of COAD (Figure 7H). The GO functional enrichment analysis demonstrated the red module was mainly enriched in BP involved in type 1 IFN signaling pathway, cellular response to type 1 IFN, antigen processing and presentation of exogenous peptide antigen via MHC class I (Figure 7I). Besides, the red module was negatively associated with the PAC2 in immune landscape (Figure 7J). Moreover, COAD patients with higher scores of genes clustered in red showed worse survival in comparison with those in lower scores (Figure 7K). Given the importance of tumor‐infiltrating immune cells in terms of therapeutic response of mRNA vaccination, the patients with higher expression of genes clustered in red module might be more appropriate for mRNA vaccination. Lastly, five immune hub genes including IFIT3, PARP9, TAP1, STAT1, and OAS2 with >95% correlation in red module were confirmed. These immune hub genes could be served as biomarkers for screening appropriate COAD patients for mRNA vaccination.

## 4 DISCUSSION

Limited by present therapeutic strategies, the prognosis of patients with advanced COAD remains unsatisfied. Therefore, developing more effective treatment modalities for patients with COAD is urgently needed. Depending on eliciting immune responses against cancer cells, cancer vaccines designed to target TAAs or TSAs are recently developing as promising strategies.^6^ The mRNA vaccines have shown superiorities compared with other vaccine approaches, such as high potency, controllable, safe administration, and rapid development. Nevertheless, published clinical trials about mRNA vaccines for treating COAD have shown limited clinical benefits.^17^, ^18^ Selecting appropriate neoantigens that are clinically relevant and immunogenic remains the key issue for developing effective mRNA vaccines.^18^ Furthermore, the complicated and immunosuppressive tumor microenvironment has posed challenges for the efficiency of mRNA vaccines. Therefore, a comprehensive analysis of the immune status is valuable for identifying the subset of COAD patients who might benefit from mRNA vaccination.

In this study, we analyzed the aberrantly expressed and mutational profiles of COAD, which is the main factor for generating neoantigens; and further predicted a wide range of potential antigens for vaccination. Considering these predicted antigens by using gene alteration profile might not be clinically relevant, the potential candidates were further narrowed through screening antigens with prognostic values. A total of five selected tumor antigens, including IGF2BP3, DPCR1, HOXD10, TRIM7, and ZIC5 were identified as promising candidates for mRNA vaccine. The higher expression levels of these antigens were not only significantly associated with the poorer prognosis of COAD, but also positively correlated with higher levels of tumor infiltrating B cells, CD8^+^ T cells, CD4^+^ T cells, and APCs. For efficiently stimulating the innate and adaptive immunity, the proteins or peptides translated from the exogenous mRNA could be presented by the APCs, mainly DCs, followed by inducing CTL through cross‐priming, as well as inducing the clonal expansion of antigen‐specific B cells via the help of CD4^+^ T cells.^42^ Therefore, the relevance between these identified antigens and tumor‐infiltrating immune cells further demonstrated their potential for developing mRNA vaccines. Although further clinical validation of these antigens is requisite, the previously published reports also supported their potential for developing mRNA vaccines. For instance, the IGF2BP3 that newly identified as N6‐methyladenosine (m6A) reader, is reported to mainly play oncogenic roles in multiple types of cancer.^43^ Overexpression of IGF2BP3 has been noticed in numerous tumor samples, and closely correlated to the progression and worse survival in COAD.^44^ Based on the lack of IGF2BP3 in most normal tissues, the IGF2BP3 has been gradually postulated as a valuable and potential target for cancer vaccine. The immunogenicity of IGF2BP3 has been confirmed by the existence of antibodies against recombinant IGF2BP3 protein in the pleural effusions from patients with lung cancer.^45^ Furthermore, the efficiency of multiple peptide‐based cancer vaccine involving IGF2BP3 peptide has been investigated by a phase II clinical trial in patients with head and neck squamous cell carcinoma, which revealed a relationship between the immune response induced by this vaccine and better prognosis.^46^ Therefore, these findings further confirm the reliability of these predicted antigens for developing mRNA vaccines.

Based on the complex tumor immune status and functional pattern of mRNA vaccine, not all the patients with COAD could benefit from the vaccination. Therefore, patients with COAD were stratified into five immune subtypes based on the immune‐related gene profiles in order to screen appropriate subset for the optimal application of vaccination. Distinct clinical characteristics have been observed among these five subtypes. Patients in IS3 and IS4 were associated with worse prognosis compared with other subtypes, indicating the prognostic value of the immunotyping in patients with COAD. Furthermore, higher TMB and somatic mutation rates were observed in IS1 and IS5 subtypes. Tumor with high TMB has relatively higher potential to generate immunogenic neoantigens.^47^ As an important component of the tumor immunogenicity, the higher TMB is tightly associated with the increased response rates in patients accepted with immunotherapy.^48^ Therefore, the patients in IS1 and IS5 subtypes with high TMB might well have better responsiveness to mRNA vaccination. Currently, single application of cancer vaccines has not achieved the desired effect on eradicating tumors.^18^ Increasing studies have shown the evidence of the cancer vaccine‐induced intratumoral infiltration of T cells, suggesting the possibility of a combination of mRNA vaccine and immunotherapies like checkpoint blockade.^49^ Thus, the expression levels of ICPs and ICD modulators were explored in the five immune subtypes. The IS1 and IS5 subtypes showed relatively elevated expression levels of ICD modulates, suggesting greater potential of mRNA vaccination might well be found in these two subtypes. Interestingly, the IS2 subtype displayed obviously higher expressions of most ICPs‐related genes, while the IS1 and IS5 subtypes showed relatively lower expressions of the ICPs‐related genes. Hence, the patients in IS1 and IS5 with relatively lower expressions of most ICPs‐related genes might be more suitable for mRNA vaccination, while the patients in IS2 with significantly highest expressions of ICPs‐related genes might have better responses to the combination therapy of mRNA vaccine and immune checkpoint inhibitors. The new combination of cancer vaccines and immune checkpoint inhibitors is becoming a promising strategy to synergistically facilitate the success of cancer treatment.^6^ Collectively, the immunotyping of COAD in this study not only has shown the prognostic relevance, but also could serve as an indicator to choose suitable patients for mRNA vaccines or combination therapies.

There's increasingly notice that clinical benefits from immunotherapies largely depend on the pre‐existing inflammation in tumor microenvironment. The immune cell components in various immune subtypes were analyzed. The IS1 and IS5 subtypes showed significantly higher enrichment scores in DCs, macrophages, CD8^+^ T cells, cytotoxic cells, CD56^bright^ NK cells, and Th1 cells in comparison with those in IS3 and IS4 subtypes. Hence, the IS1 and IS5 subtypes that characterized with the baseline of immune‐hot tumor microenvironment, while the IS3 and IS4 subtypes displayed relatively non‐inflamed (immune‐cold) phenotypes. The patients who showed better responses to immunotherapies tend to have immune‐hot tumors that characterized by abundant infiltration of immune cells. Previous study has shown that the higher immune cell infiltration scores in patients with head and neck squamous cell carcinoma were significantly correlated with better objective responses to immunotherapies including immune checkpoint inhibitors, cytokines, and vaccines.^50^ In melanoma, the inflamed tumor microenvironment such as T‐cell marker that existed before vaccination was associated with good clinical responses.^51^ Inspired by these results, the following study has analyzed the immune gene profiles of the pre‐treatment tumor biopsies from a second vaccine trial, which revealed a positive association between inflamed gene expression profiles and clinical benefits from a DC‐based vaccine.^52^ Furthermore, a clinical trial that validating the effectiveness of therapeutic cancer vaccine Theratope in patients with ovarian, breast or colorectal cancer demonstrated the correlations between pre‐existing inflammation and clinical benefits. However, the patients were not screened or stratified according to their pre‐vaccination immune status in the subsequent phase III clinical trial.^41^ It's extremely rewarding to integrate the pre‐existing inflammation as a filtration or stratification factor in the further immunotherapy trials. Moreover, the direct injection of mRNA vaccines into tumor sites might well empower a rapid activation and expansion of pre‐existing T cells.^42^, ^53^ Therefore, the COAD patients in IS1 and IS5 subtypes characterized with elevated immune cell scores might benefits more from the mRNA vaccination. The combination of other immunostimulatory therapies with mRNA vaccination to boost the weak antitumor immunity might well be better approaches for COAD patients in the subtypes with relatively low immune scores.

Moreover, the heterogeneity of individual patients even within the same immune subtype was revealed by the analysis of immune landscape, which were conducive to narrow the immune components for better personalized mRNA‐based vaccination. The IFIT3, PARP9, TAP1, STAT1, and OAS2 were confirmed as the hub genes in red module, which negatively related to PCA2 of the immune landscape. Therefore, the COAD patients with high expressions of these genes might be more appropriate for mRNA vaccination.

In conclusion, this study identified 5 potential targets for the development of mRNA vaccines against COAD, including IGF2BP3, DPCR1, HOXD10, TRIM7, and ZIC5. The patients were further stratified into 5 immune subtypes according to immune‐related gene profiles, among which the patients in IS1 and IS5 subtypes might respond better to mRNA vaccination. Our study not only offers a theoretical foundation of developing mRNA vaccines and screening appropriate COAD subsets for vaccination, but also provides a scientific method for identifying potential antigens of vaccines in other cancer types or even pandemics like COVID‐19.

## AUTHOR CONTRIBUTIONS

H.T. performed the literature search and bioinformatics analysis; T.Y. participated in the bioinformatics analysis and wrote the original draft; C.L., Y.W., Z.D. and J.L. helped with the data collection and analysis; C.L. and F.J. helped with data analysis, and revised the manuscript; H.S. conceived the study, helped with data analysis and interpretation, and revised the manuscript. All the authors have read and approved the final manuscript.

## FUNDING INFORMATION

This work was supported by National Natural Science Foundation of China (82003195), the China Postdoctoral Science Foundation (2020 M680150), and Post‐doctoral research project, West China Hospital, Sichuan University (2020HXBH002).

## CONFLICT OF INTEREST

The authors declare that the research was conducted in the absence of any commercial or financial relationships that could be construed as a potential conflict of interest.

## ETHICS STATEMENT

All data of this study were public and required no ethical approval.

## Supporting information


Figure S1–S7

Table S1

## Data Availability

All data generated and described in this article are available from the corresponding web servers, and are freely available to any scientist wishing to use them for noncommercial purposes, without breaching participant confidentiality. Further information is available from the corresponding author on reasonable request.
