# Supplementary material for: Identifying tumor antigens and immuno‐subtyping in colon adenocarcinoma to facilitate the development of mRNA vaccine
Source: Cancer Med. 2022 May 20;11(23):4656–72. doi: 10.1002/cam4.4846 (PMC9741973; doi:10.1002/cam4.4846)
Supplement: Supplementary file 1 — Figure S1–S7 [file CAM4-11-4656-s001.docx]

**Identifying tumor antigens and immuno-subtyping in colon adenocarcinoma to facilitate the development of mRNA vaccine**

Huaicheng Tan^1^, Ting Yu^2^, Chunhua Liu^1^, Yang Wang^1^, Fangqi Jing^3^, Zhenyu Ding^1^, Jiyan Liu^1^, Huashan Shi^1, 4^

^1^ Department of Biotherapy, Cancer Center, West China Hospital, Sichuan University

^2^ Department of Pathology and Laboratory of Pathology, State Key Laboratory of Biotherapy, West China Hospital, West China School of Medicine, Sichuan University

^3^ State Key Laboratory of Oral Diseases, National Clinical Research Center for Oral Diseases, West China Hospital of Stomatology, Sichuan University, Chengdu 610041, China

^4^ Department of Radiotherapy, Cancer Center and State Key Laboratory of Biotherapy, West China Hospital, Sichuan University

**Correspondence:** Huashan Shi; E-mail address: [shihuashan@scu.edu.cn](mailto:shihuashan@scu.edu.cn); Tel: +8618980606519; Fax: +2886621955.

Huaicheng Tan, and Ting Yu contributed equally to this work.

**Funding:** This work was supported by National Natural Science Foundation of China (82003195), the China Postdoctoral Science Foundation (2020M680150) and Post-doctoral research project, West China Hospital, Sichuan University (2020HXBH002).

**Supplementary Figure**

**
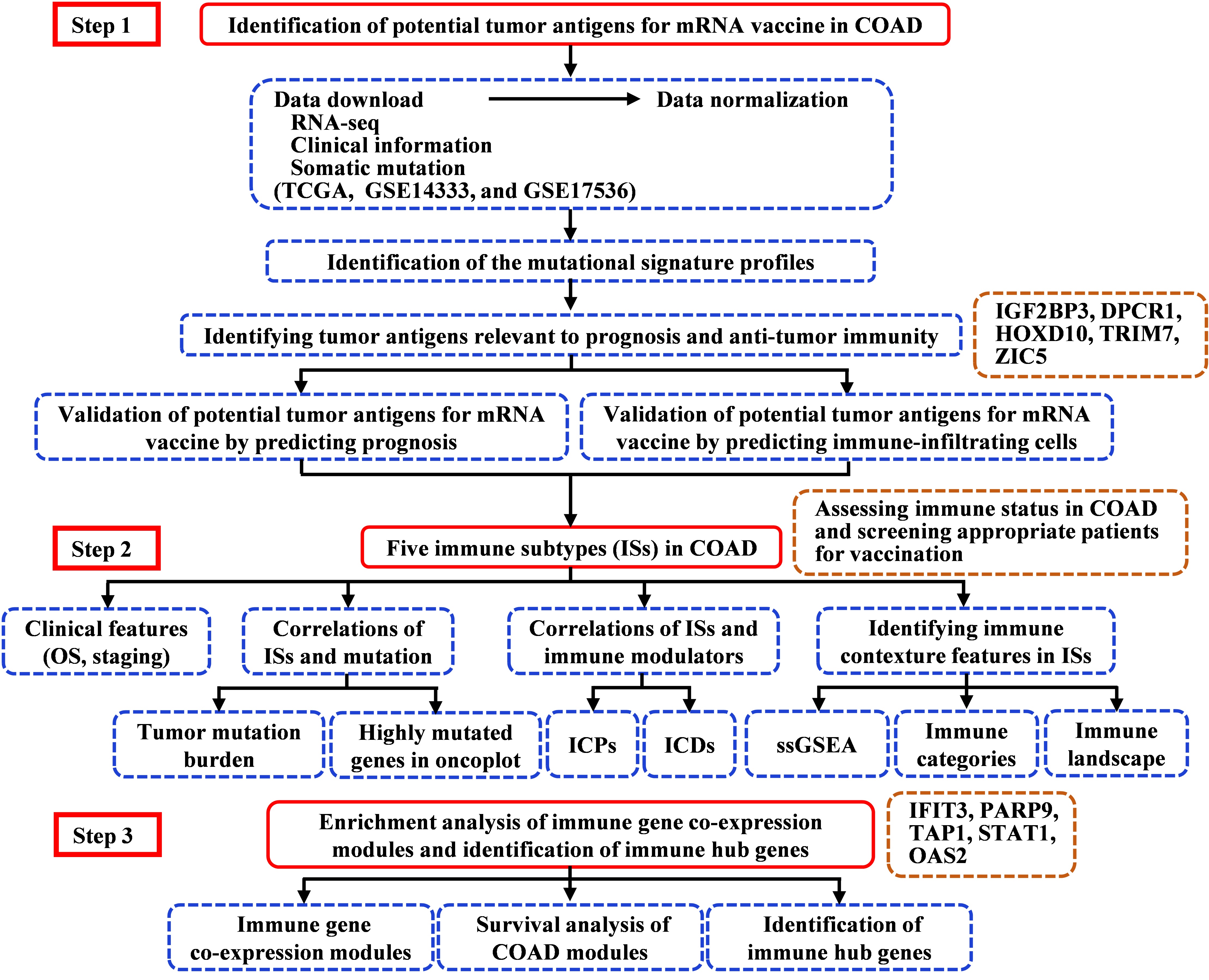
**

**Figure S1.** Flow diagram of the data processing, analysis, and validation in this study.

**
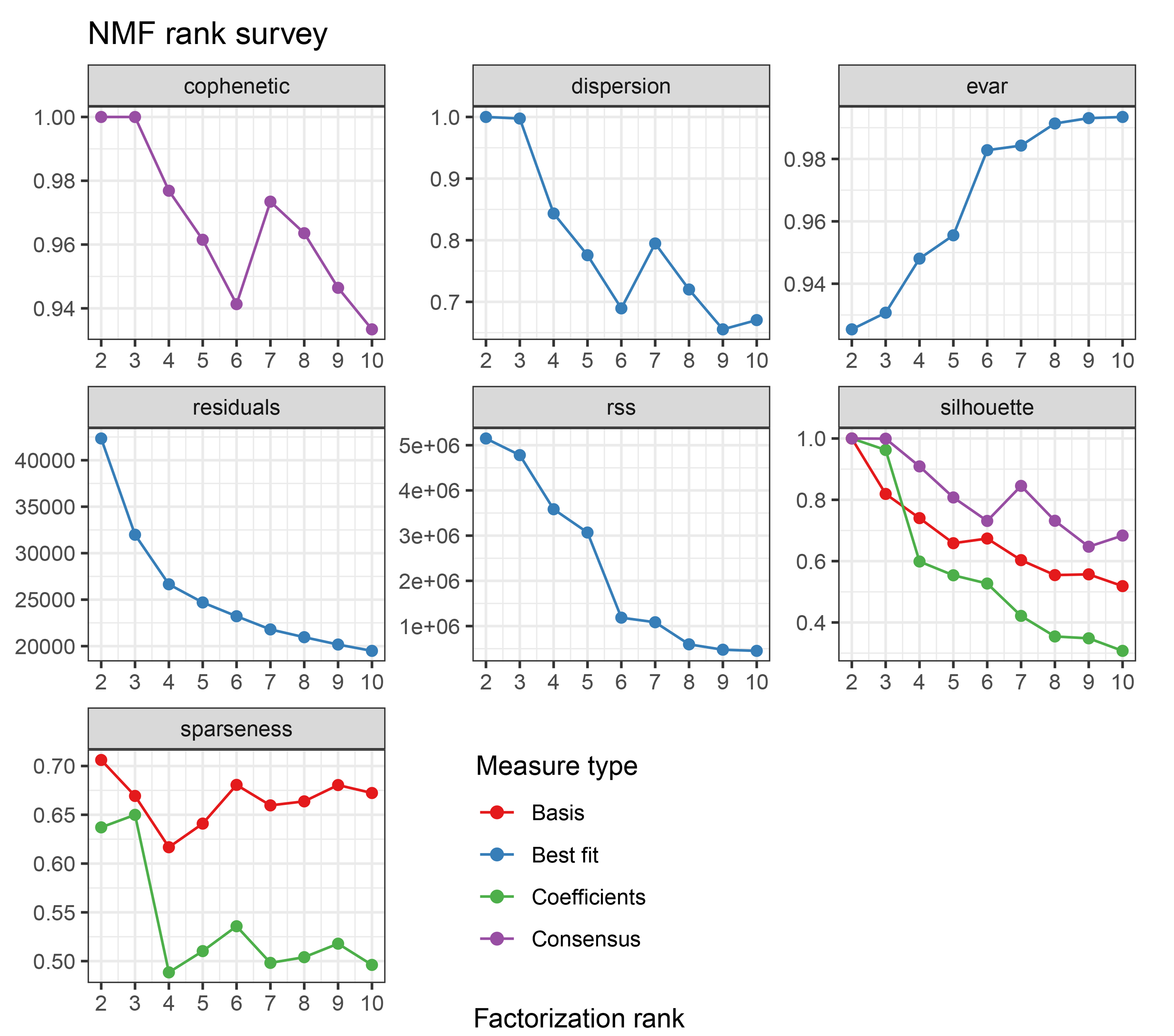
**

**Figure S2.** Rank survey of the parameter *r* in NMF.


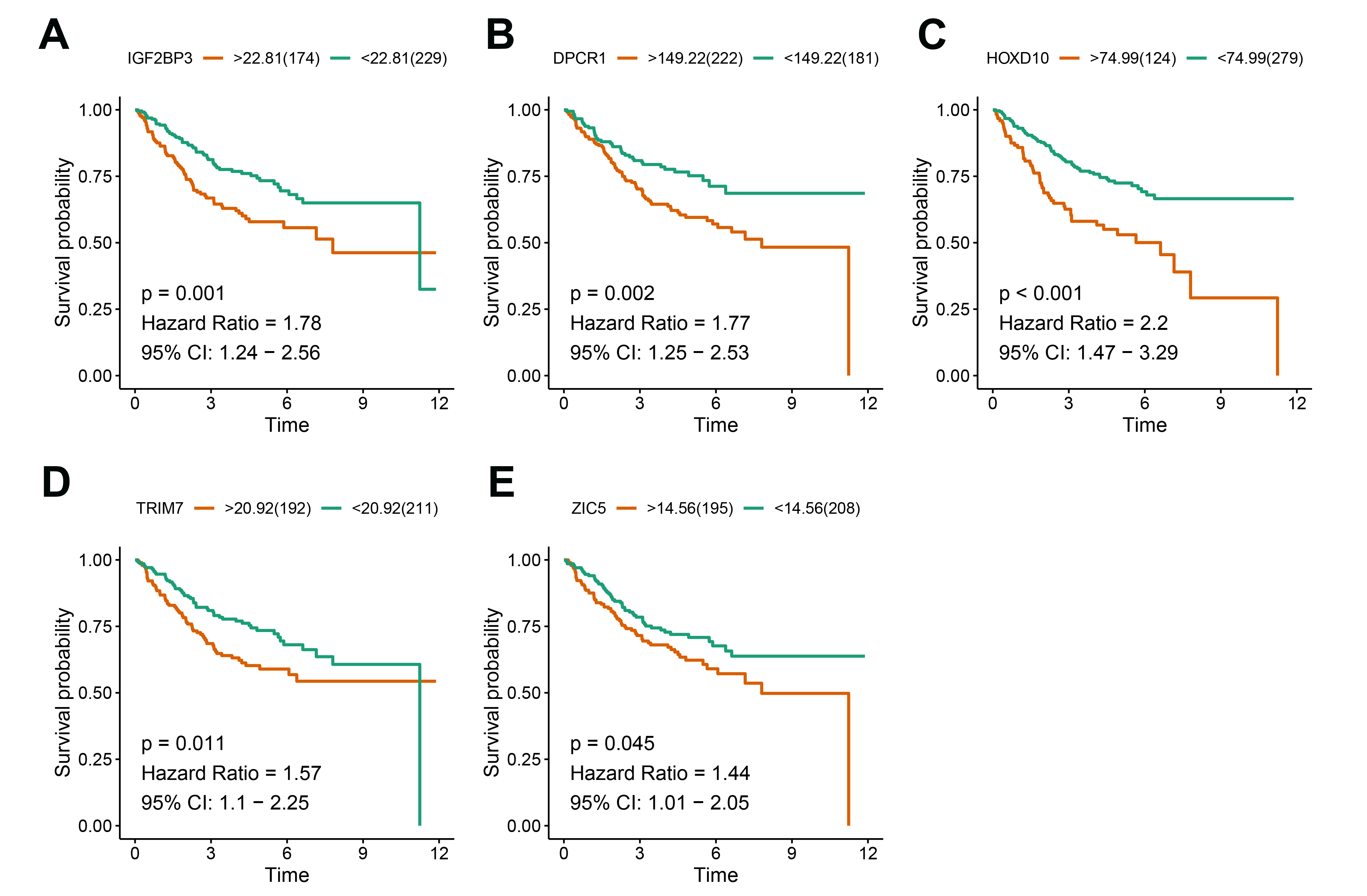


**Figure S3.** Prognostic relevance of the potential tumor antigens in GSE14333 and GSE17536. The higher expression levels of candidate genes for mRNA vaccine, including IGF2BP3 (A), DPCR1 (B), HOXD10 (C), TRIM7 (D), and ZIC5 (E) were associated with worse OS in COAD patients, as calculated by the Kaplan-Meier analysis.


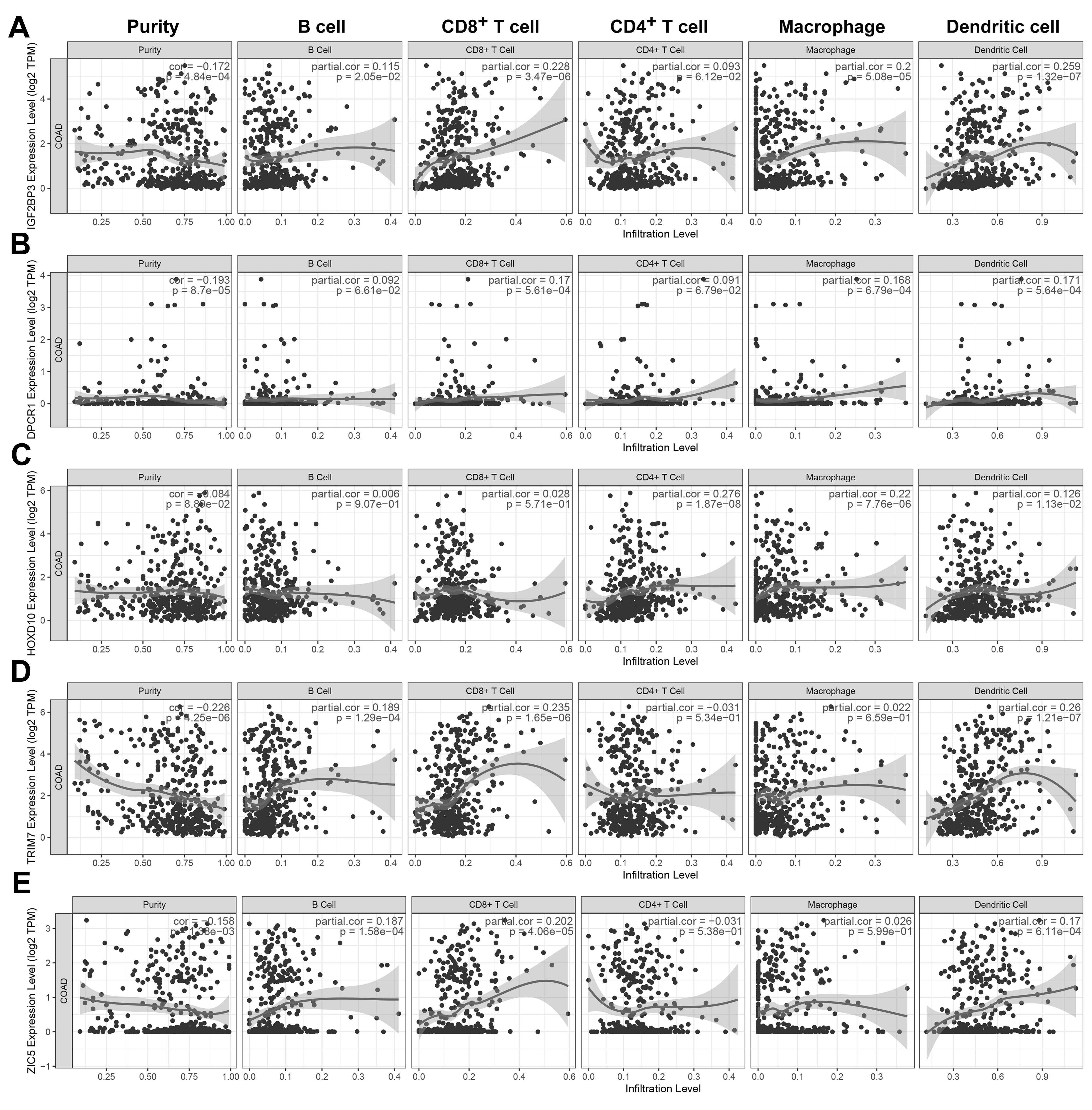


**Figure S4.** The expression levels of identified tumor antigens tended to be positively related to the intratumoral infiltration of immune cells. The correlations between the expression levels of IGF2BP3 (A), DPCR1 (B), HOXD10 (C), TRIM7 (D), and ZIC5 (E) and the intratumoral infiltration of B cells, CD8^+^ T cells, CD4^+^ T cells, macrophages and DCs in COAD patients were analyzed by TIMER.


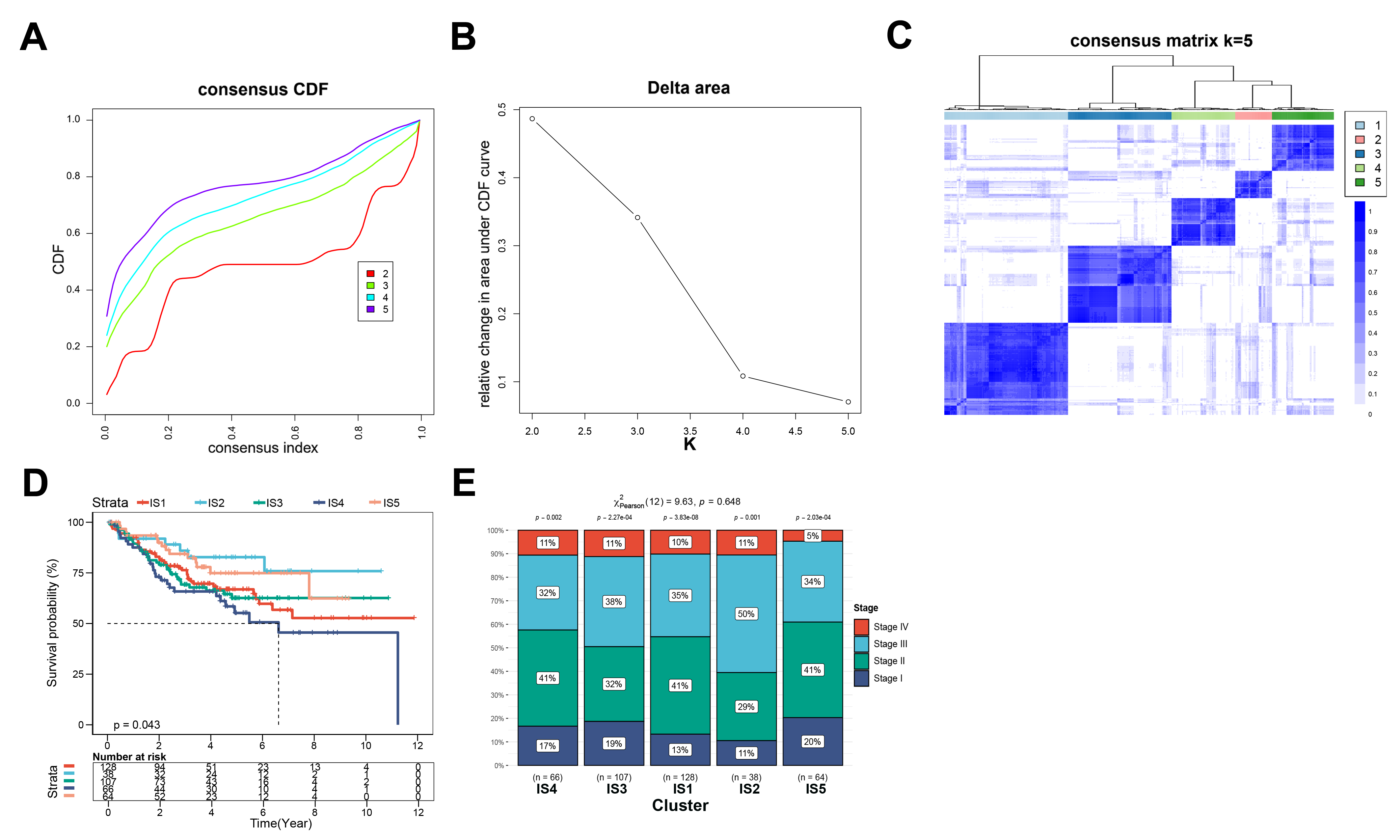


**Figure S5.** Immunosubtyping of the COAD patients from datasets of GSE14333 and GSE17536. The CDF curves (A) and delta area of immune-related genes (B) were analyzed. (C) Clustering heat map of COAD based on immune-related gene expression profile. (D) OS analysis of COAD patients with different immune subtypes. (E) Staging distribution in COAD patients with IS1-IS5.


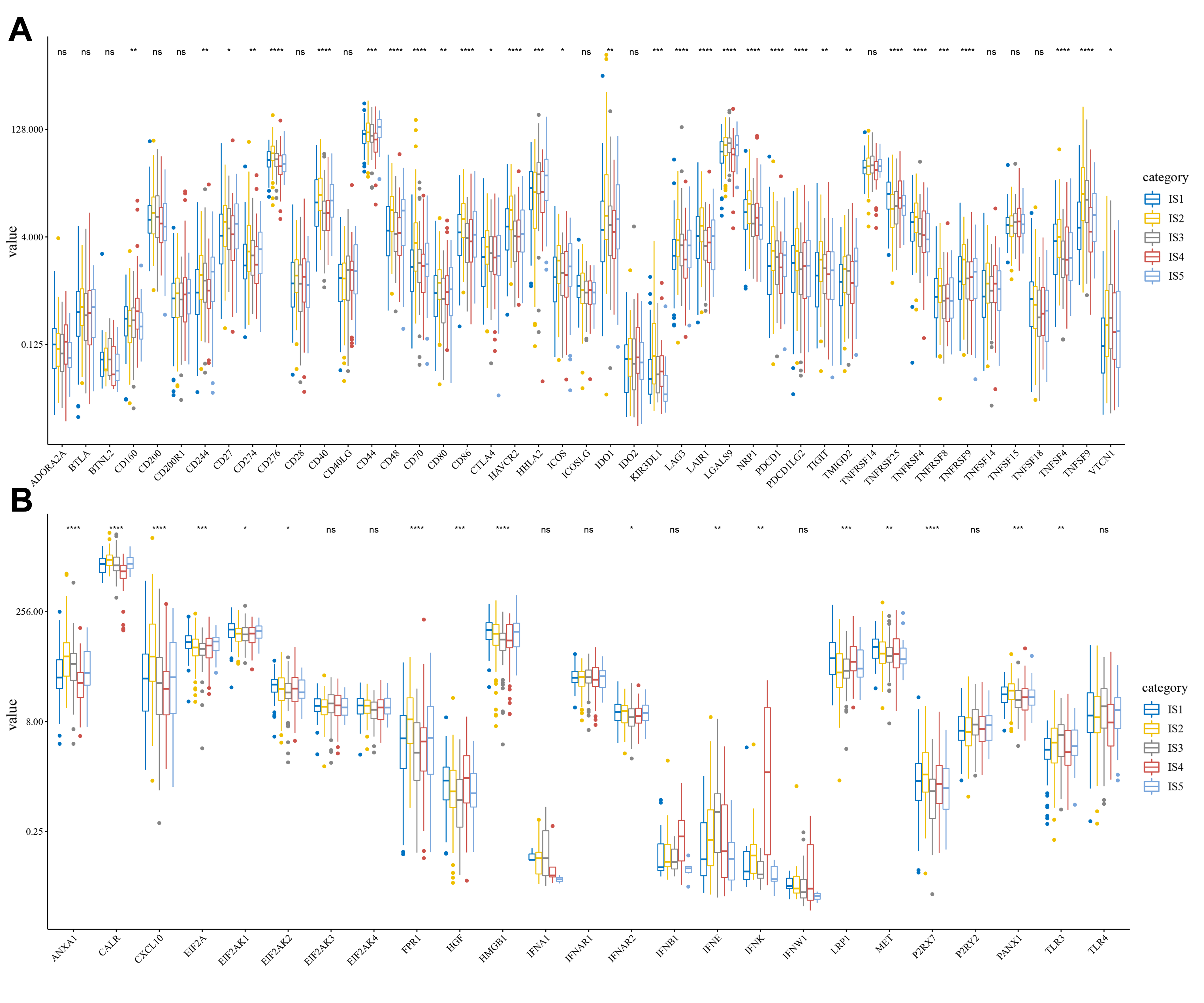


**Figure S6.** Expression status of the ICPs and ICD modulators among various immune subtypes in TCGA cohort. Evaluating the expression levels of representative ICP genes (A) and representative ICD genes (B) in COAD immune subtypes. (*P < 0.05, **P < 0.01, ***P < 0.001 and ****P < 0.0001).


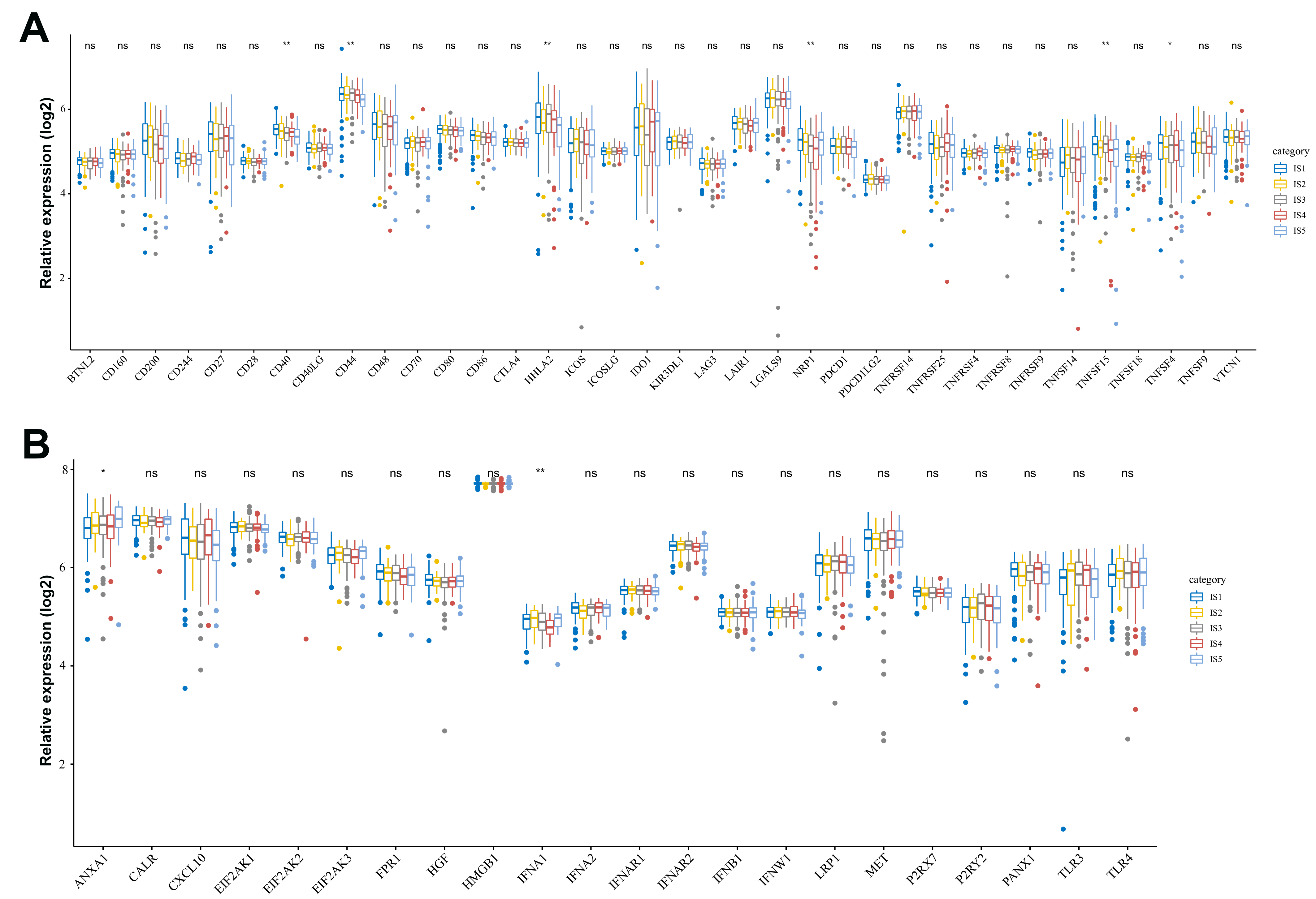


**Figure S7.** Expression status of the ICPs and ICD modulators among various immune subtypes in the datasets of GSE14333 and GSE17536. Evaluating the expression levels of representative ICP genes (A) and representative ICD genes (B) in COAD immune subtypes. (*P < 0.05, and **P < 0.01).
